# Predicting Single Neuron Responses of the Primary Visual Cortex with Deep Learning Model

**DOI:** 10.1002/advs.202305626

**Published:** 2024-02-13

**Authors:** Kaiwen Deng, Peter S. Schwendeman, Yuanfang Guan

**Affiliations:** ^1^ Department of Computational Medicine and Bioinformatics University of Michigan Ann Arbor MI 48105 USA; ^2^ College of Engineering University of Michigan Ann Arbor MI 48105 USA

**Keywords:** deep learning, neuron responses prediction, primary visual cortex

## Abstract

Modeling neuron responses to stimuli can shed light on next‐generation technologies such as brain‐chip interfaces. Furthermore, high‐performing models can serve to help formulate hypotheses and reveal the mechanisms underlying neural responses. Here the state‐of‐the‐art computational model is presented for predicting single neuron responses to natural stimuli in the primary visual cortex (V1) of mice. The algorithm incorporates object positions and assembles multiple models with different train‐validation data, resulting in a 15%–30% improvement over the existing models in cross‐subject predictions and ranking first in the SENSORIUM 2022 Challenge, which benchmarks methods for neuron‐specific prediction based on thousands of images. Importantly, The model reveals evidence that the spatial organizations of V1 are conserved across mice. This model will serve as an important noninvasive tool for understanding and utilizing the response patterns of primary visual cortex neurons.

## Graphical abstract

This study presents a computational model that advances the prediction of single neuron responses to natural stimuli in the mouse primary visual cortex (V1) by 15%–30% with the incorporation of object positions and multiple model ensembles. It computationally reveals the neuron properties and spatial organizations across multiple mice, offering a noninvasive tool for studying the primary visual cortex activity.

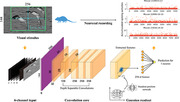

## 1 Introduction

Accurate predictive models of neural population activity have emerged as invaluable tools to help bridge the gap between biological and computational understandings of the visual system.^[^ ^1^, ^2^ ^]^ Acting as digital twins of the visual cortex, these models allow neuroscientists to propose and test hypotheses both in silico and in vivo^[^ ^3^, ^4^ ^]^ and can inform the biomedical engineering of modern AI visual assistants.

Predictive modeling of neuronal visual responses has a storied history, featuring algorithms that range from simple linear‐nonlinear models^[^ ^5^ ^]^ to multi‐layer perceptron models^[^ ^6^ ^]^ to contemporary deep‐learning‐based algorithms.^[^ ^7^, ^8^, ^9^, ^10^ ^]^ Broadly, there are two types of approaches to learning the complex nonlinearity in neuron responses: task‐driven methods and data‐driven methods. Task‐driven methods rely on transfer learning concepts, predicting single neuron responses using features pretrained on other computer vision tasks.^[^ ^7^, ^8^, ^11^ ^]^ In contrast, data‐driven methods infer thousands of neurons from the shared features between subjects and train an entire network end‐to‐end on stimulus‐response pairs.^[^ ^12^, ^13^ ^]^ A recent study reported that pretraining weights offered little benefit, and the resulting optimized networks might fail to capture the cortex's visual hierarchy.^[^ ^14^ ^]^ Consequently, the current state‐of‐the‐art model for mice was built on the data‐driven approach with a core‐readout framework.^[^ ^15^ ^]^

We are interested in investigating two questions in this study. First, can we develop a better methodology to predict an individual neuron's response based on the current frameworks? With such a method, we will have a tool to dissect the visual cortex in silico. Second, what can we understand about the behavior and organization of the visual cortex through such a predictive tool? A prediction algorithm can serve as an abstraction of the principles behind neural responses. To answer these questions, we leveraged the SENSORIUM challenge data, which offers benchmarks to expedite the search for the optimal predictive model for the primary visual cortex (V1) in mice. This dataset recorded neuron responses from seven mice to thousands of natural images and simultaneous behavioral measurements such as pupil position and dilation.^[^ ^16^ ^]^ We developed an algorithm that ranked first in the stimulus‐only track (excluding inputs from eye behaviors), representing a significant leap from existing models.

Our method's main contributions include integrating object position information and applying an ensemble strategy in training. These enhancements propelled our model to outperform the baseline by almost 15% on average. Apart from the computational significance, our model consistently mirrored biological patterns observed during in vivo studies, such as the mechanisms of processing visual inputs and the spatial properties of the primary visual cortex (V1).^[^ ^17^, ^18^, ^19^ ^]^ It also suggested that V1 would react similarly to the same stimulus across mice. As our model provides more accurate and biologically meaningful predictions for single‐neuron behaviors under any image stimuli, it is capable of serving as a noninvasive tool that will not only help neuroscientists better understand the principles of the primary visual cortex^[^ ^4^, ^20^ ^]^ but also have the potential to control neurons more precisely and enlighten a new class of therapeutic applications.^[^ ^21^ ^]^

## 2 Results

### 2.1 Overview of the Experiment Design for Predicting Sensory Neural Responses to Natural Stimuli

**Figure 1:**
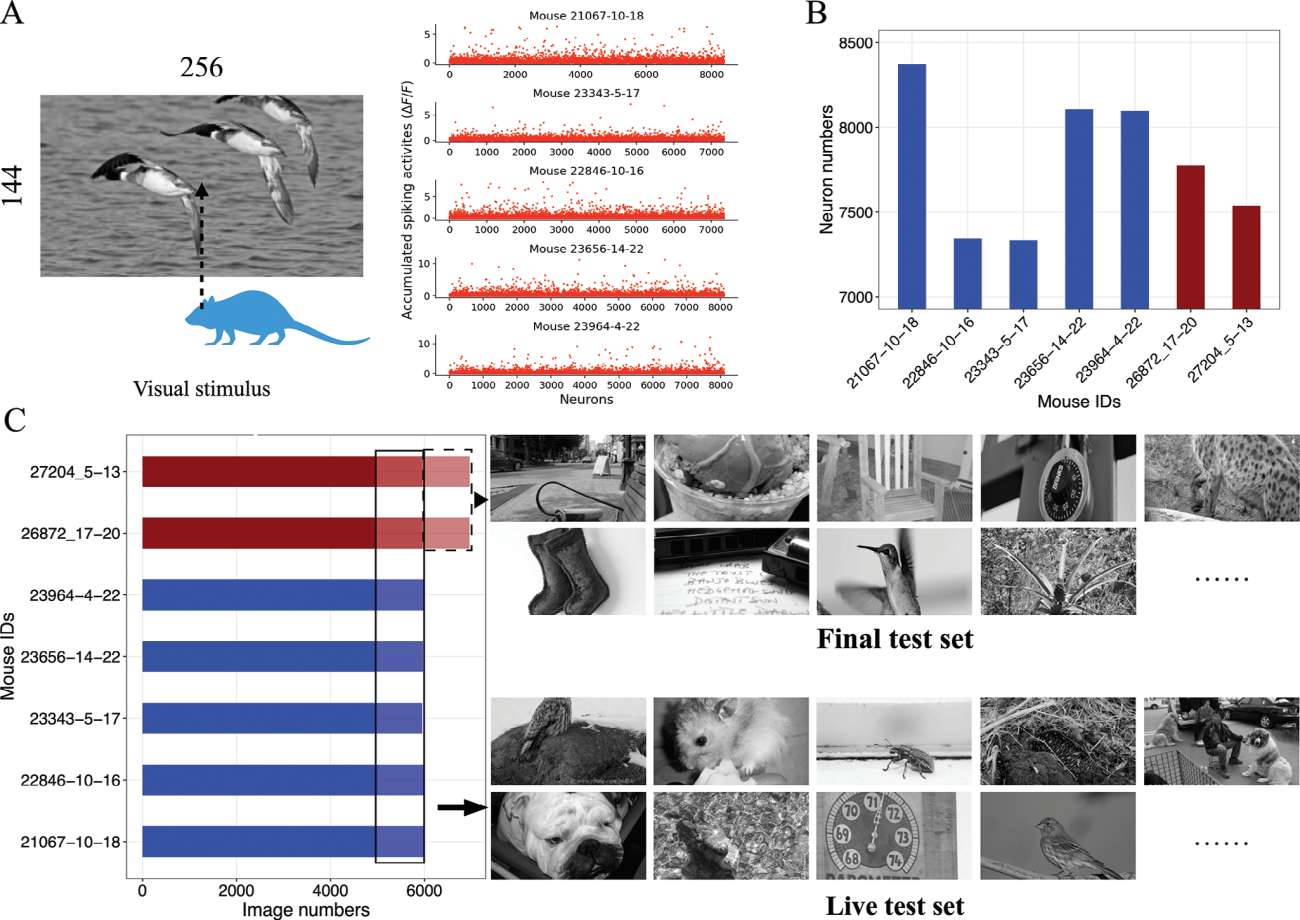
Experimental design and data of the Sensorium competition stimulus‐only track. A) visualizes a live test example pair of visual stimulus and neuron responses from the five mice for pretraining in the SENSORIUM challenge. The stimulus is a grayscale image with a size of 144 in height and 256 in width. The corresponding responses are accumulated spiking activities in relative fluorescence changes (Δ*F*/*F*) retrieved from the calcium images using the CAIMEN pipeline. The signal for each neuron was recorded in a single value. B) shows the neuron numbers of each mouse. The mice with red bars are the held‐out test sets, and the “26 872_17–20” was for the stimulus‐only track. C) shows the number of stimuli for each mouse. Examples of the images in live and final test sets are also listed.

The data from the Sensorium 2022 competition that we used to develop our model comprised pairs of excitatory neuron responses and visual stimuli from ImageNet in seven mice (**Figure** **1**A). Experimenters recorded neuron activities within layer 2/3 of the right primary visual cortex (V1), responding to 43 805 visual stimuli and covering 25200 unique instances. The stimuli were grayscale images cropped into a 16:9 aspect ratio with the dimensions of 256‐by‐144 pixels. Each was presented to the mice for 500 ms, followed by a black screen period between 300 and 500 ms. Neuron signals (i.e., the spiking activities) were collected through the two‐photon calcium images and measured as the relative fluorescence changes (Δ*F*/*F*) from the images. Δ*F* denoted the difference between neuron and baseline signals and *F* served as the background. They were generated with the CAIMAN pipeline^[^ ^22^ ^]^ and accumulated between 50 and 550 ms after each stimulus onset using a boxcar window^[^ ^16^ ^]^ to form the neuron responses. For a specific neuron *i*, the neuronal response to a stimulus was represented by a single value *r_i_* (Figure 1A, accumulated Δ*F*/*F*). The number of neurons recorded varied among mice, ranging from 7334 to 8372 (Figure 1B). In addition, the anatomical coordinates of each neuron relative to the experimenter's estimate of the pial surface were recorded along with the calcium responses.

During the challenge, data from two mice were designated as held‐out competition test sets, while the others served as pre‐training datasets for model refinement. Each dataset contained a subset of 100 unique images for live testing, enabling real‐time model building and leaderboard updates throughout the submission period. An additional 100 unique images were exclusively included in the held‐out competition sets for final testing and determining the winning entries (Figure 1C). These test images were repeated ten times and randomly interspersed with other images during neuronal response measurements. The two held‐out datasets' live and final test responses will never be publicly released.

**Figure 2:**
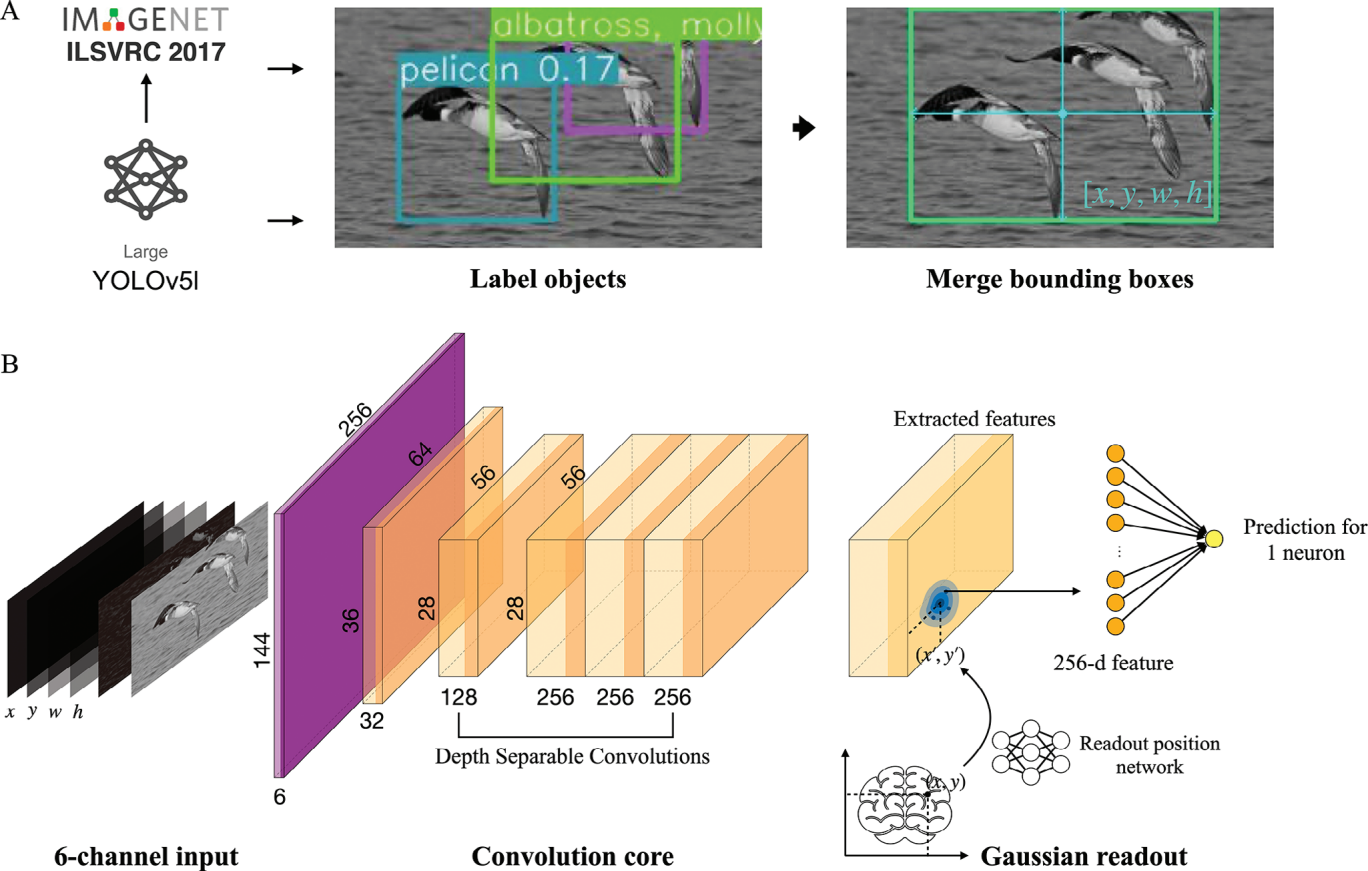
Schemata of the method. A) describes the method for extracting the object positions from the images. The official YOLOv5 large model from PyTorch, which has already been pre‐trained on COCO, is used for detecting humans. We fine‐tuned it on the ILSVRC dataset for detecting other types of objects. The bounding boxes will then be merged and expanded to a larger one to cover all the detected objects. B) is the model structure. The model encodes the six‐channel input through the convolution core and decodes the extracted features to the single neuronal predictions with a Gaussian readout.

Our model was developed with several significant enhancements over the current state‐of‐the‐art data‐driven method.^[^ ^23^ ^]^ One crucial feature involved incorporating image object information as additional input channels. This is based on the assumption that the neuronal response may follow an attention mechanism focusing on the object in the image.^[^ ^7^, ^24^, ^25^ ^]^ To identify the object, we fine‐tuned an object detection model based on the YOLOv5 large network^[^ ^26^ ^]^ on 250 000 gray‐scaled images from the ImageNet Large Scale Visual Recognition Challenge (ILSVRC) dataset,^[^ ^27^ ^]^ achieving a mean average precision (mAP) of 0.5 on its validation set with 50 000 images. For each image, the detected object bounding boxes were merged and padded to cover all objects with one box, enabling us to extract the width (w), height (h), and coordinates of the box center (x, y) for each image (**Figure** **2**A). To train the model, we constructed a 6‐channel input including the (*x, y, w, h*) object information, the image normalized by the global averages and standard deviations of the training and validation images of the current dataset, and the image centered by subtracting the average of itself (Figure 2B, see Methods). We then employed an ensemble strategy, another key feature of our solution, by training multiple models on different train‐validation splits and averaging their predictions to calculate our final outputs.

### 2.2 The Object‐Integrated Ensemble Model Significantly Improved the Accuracy of the Cross‐Subject Prediction

**Figure 3:**
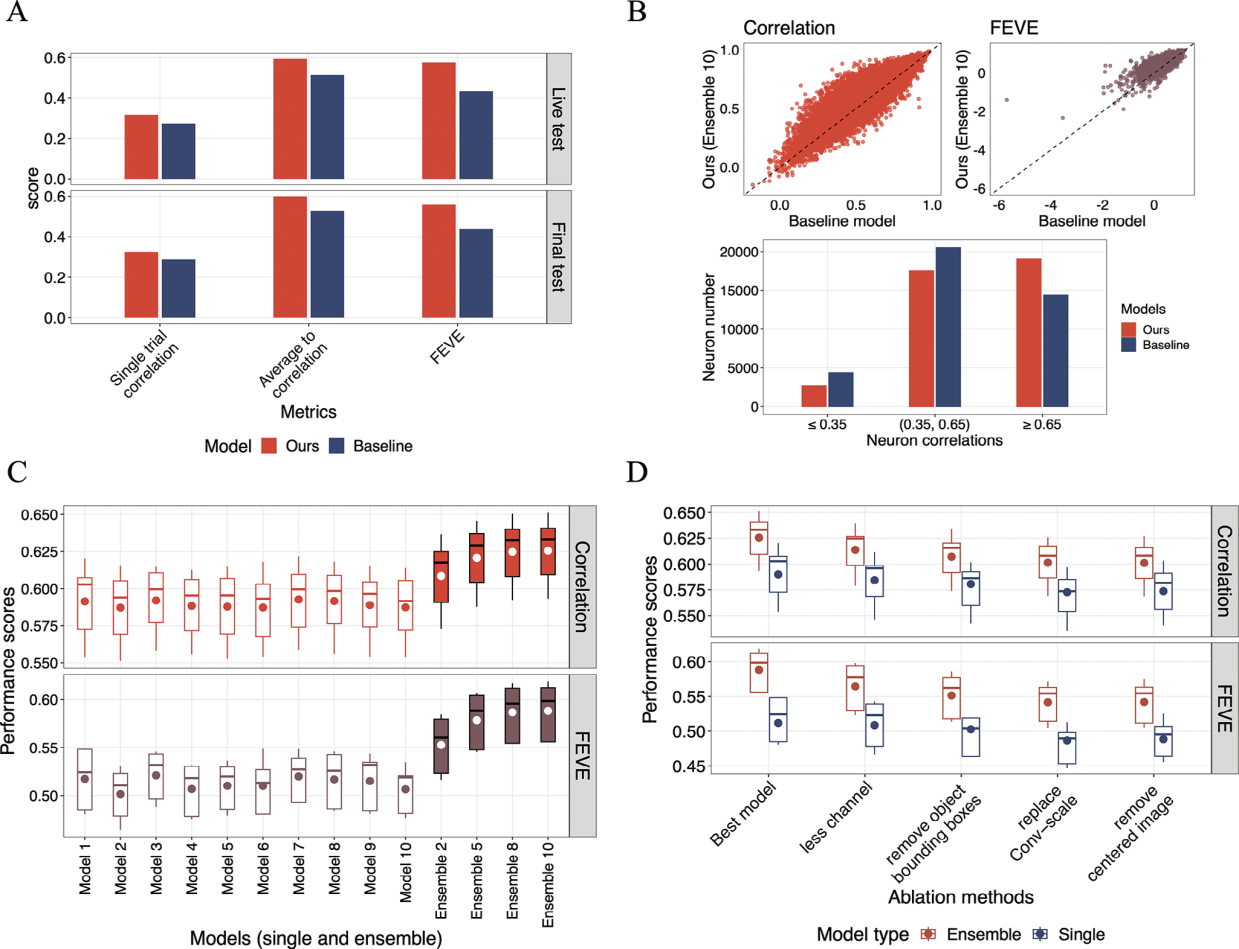
Model performances in the life and the final test set and the ablation studies. A) shows the performance of our solution compared to the baseline in the held‐out test mouse. B) compares performances between the baseline model and our method at the single‐neuron level. The top two panels show the correlation and FEVE results. The bottom panel compares the correlation score distributions with the baseline model. C) compares the ensemble models and the 10 single models before assembling. The boxplots with “Model” on the x‐axis show the single models' performances. The boxplots with “Ensemble” are the performances of assembling different numbers of single models. For example, “Ensemble 5” means we assemble the single models from “Model 1” to “Model 5” D) summarizes the ablation study results. The x‐axis label, starting from “Best model”, indicates what is removed from the previous model.

To evaluate the neuron‐wise performance of the model and rank the entries for the stimulus‐only track, the challenge proposed two metrics: the Correlation to Average ($\bar{R}$) and the Fraction of Explainable Variance Explained (FEVE). $\bar{R}$ first computed the correlation for each neuron between the prediction and the average neuronal responses across repeated presentations of the same stimuli, then averaged each individual correlation value across neurons. FEVE computed the ratio between the explained variance by the model and the explainable variance in neuronal responses for each neuron and then averaged across them.^[^ ^7^ ^]^ Our model produced robust results for the neurons in the held‐out mouse, achieving 0.594 and 0.600 $\bar{R}$ in the live test set and the final test set images, outperforming the baseline by 15.79% and 13.64%, respectively. The corresponding FEVE scores were 0.576 and 0.558, marking a substantial improvement of 33.02% and 27.62% over the baseline (**Figure** **3**A).

As the ground truths for the held‐out mouse were concealed, to closely inspect the model performance and quantify the contributions of our method, we used the metrics results on the 5 pre‐training mice. We compared the performances at the single‐neuron level. Among the 39255 neurons of the 5 mice, 77.12% had improved $\bar{R}$ with an average improvement of 0.050 (±0.074), and 79.89% had improved FEVE scores with an average improvement of 0.098 (±0.146). More neurons were well‐explained by our method. 48.51% of neurons had the $\bar{R}$ exceeding 0.65, 44.72% fell between 0.35 and 0.65, and a mere 6.77% scored below 0.35. The proportions in the baseline model were 36.61%, 52.28%, and 11.11% (Figure 3B).

Our performance gains can be attributed to two key factors: the ensemble strategy and modifications to both input data and the model structure. Ensemble approximated the improvements when training the model with more data. Compared to the single model, an ensemble with 5 models improves the performance by 5.07% (±0.85%) for correlation scores and 12% (±1.43%) for FEVE. However, increasing the number of models to ten might be inefficient in practice, as repeating the entire training process was time‐consuming, and the improvements were minimal (0.81% ± 0.15% for correlation scores and 1.55% ± 0.29% for FEVE, Figure 3C).

Modifications to data and structure, on the other hand, improved performance by providing extra prior knowledge and enhancing the model's ability to extract meaningful information from the data. With an ablation study gradually removing the following enhancements: 1) increasing the channel numbers, 2) incorporating object position, 3) adding a convolutional layer for image scaling, and 4) adding the centering‐normalized image as an additional channel, we found that most modifications positively impacted both the single model and the ensemble. By adding them to the base model one by one in the reverse order, for the single models, the average improvements compared to the previous one were 0.78% (±0.65%, correlation) and 2.2% (±1.52%, FEVE), leading to overall improvements of 3.13% and 8.49% over the baseline. For the ensemble model, the average increases in correlation and FEVE scores were 1.20% (±0.77%) and 2.4% (±1.76%), respectively, resulting in overall gains of 4.86% and 9.97% (Figure 3D).

### 2.3 Image Contents and Brightnesses Influence the Model‐Predicted Neuron Activities

**Figure 4:**
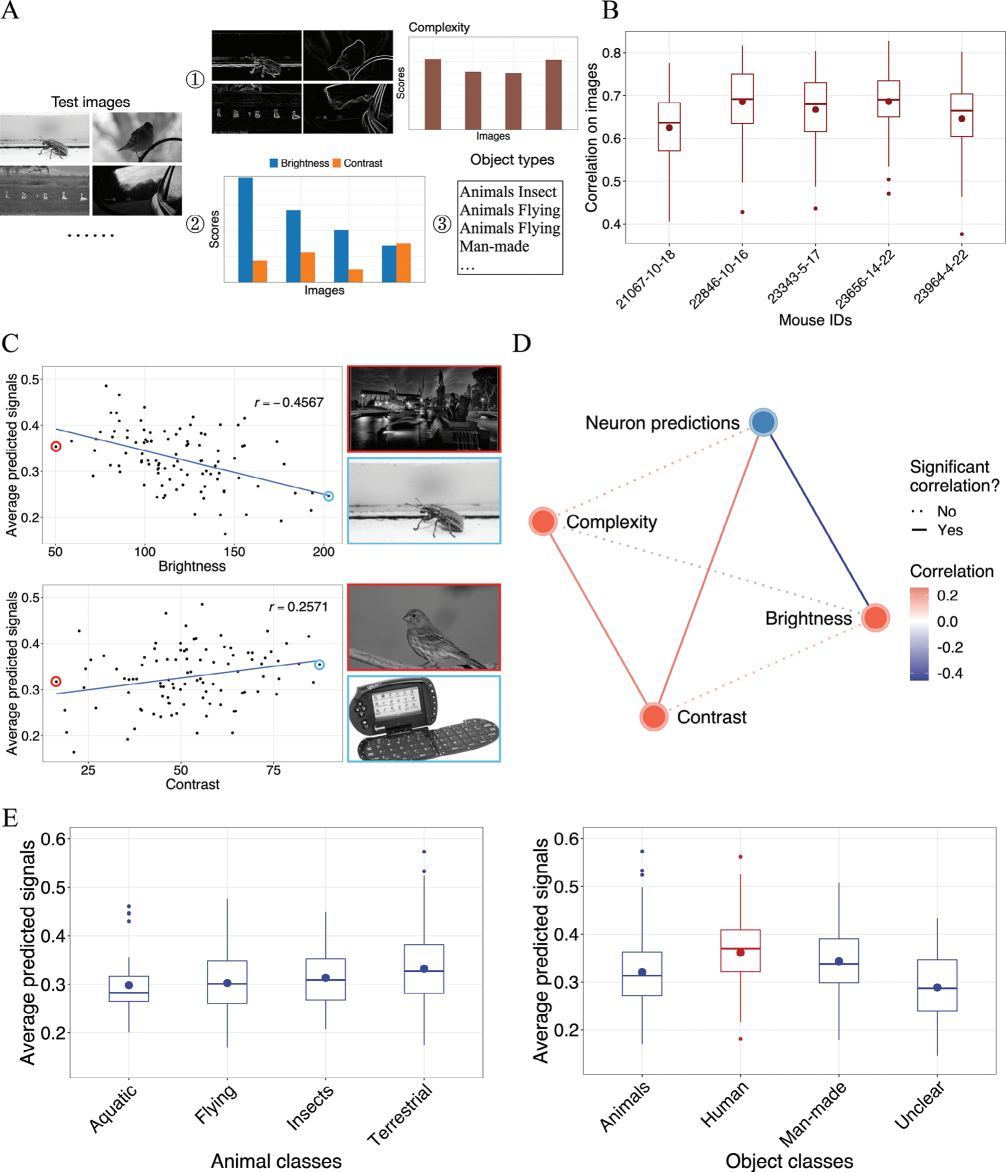
Analysis of the relationship between the model predictions and image properties. A) is a schematic representation of the image properties and contents we retrieved in this section, including the image complexity, brightness and contrast, and object classes shown in the images. We show examples to explain the complexity scores in ①. The edge images visualize the spatial information (SI) of the 4 example images (see Methods for more details), and the bar plots are the average SIs. The barplot in ② shows the brightness and contrast values of the examples. And ③ lists the object classes of these examples. B) summarizes the model performances at the image level. C) plots the influences of brightness and contrast on the predicted signals and D) draws the correlations among the image properties (brightness, contrast, complexity) and their effects on the predicted signals. The line colors are related to the correlation values, and the line types indicate whether the relationship is significant. E) shows the effects of the image contents on the predicted signals. The left panel contains different types of animals.

To test how the image properties and contents affected the model predictions, we calculated the brightnesses, contrast, and complexities of the images as the edges and luminance of stimuli were widely mentioned in previous studies as key factors affecting neuron activities.^[^ ^17^, ^28^, ^29^, ^30^ ^]^ Complexity was quantified by the average amount of spatial information, which reflects the edge magnitude of an image.^[^ ^31^ ^]^ As the analyses were between the image and the predictions, we first tested how the model performed at the image level. The correlation for each image was determined by comparing the average responses from ten repeats with the model's predictions on all neurons. The average correlation coefficients of the five mice ranged from 0.625 (±0.081) to 0.686 (±0.067) (**Figure** **4**B). This high level of accuracy underscored the validity of conclusions drawn from our predicted signals.

In comparing signal predictions with image properties, our computational model displayed biological patterns similar to those observed in prior in vivo studies. Correlating the inferred signals with image edge complexity, brightness, and contrast yielded evidence that V1 responses depend more on luminance and contrast than edge information, as previous research claimed.^[^ ^17^, ^32^ ^]^ In particular, darker (Correlation R = −0.4567, *p*‐value < 0.001) or higher contrast (Correlation R = 0.2571, *p*‐value = 0.009) images appeared to significantly activate the neurons, respectively (Figure 4C,D). The asymmetric predictions with respect to the luminances showed consistent patterns with the light‐dark cortical asymmetry of V1.^[^ ^33^, ^34^ ^]^ In contrast, the impact from edges (complexity) was comparatively moderate (Correlation R = 0.1767, *p*‐value = 0.0785, Figure 4D, Figure S2A, Supporting Information). Moreover, inspection of the effects of luminance and contrast might suggest that these properties affect the neuron activities independently: they had a moderately positive correlation (Correlation R = 0.1520), but their influences on neuron activity were inverse (Figure 4D, Figure S2A, Supporting Information).

We then manually categorized the images based on the depicted objects: animal (flying, terrestrial, aquatic, insects), human, man‐made, and an “unclear” category for hard‐to‐identify objects (Figure 4A, Figure S1, Supporting Information). The strength of neuronal signals was influenced by the contents of the images. Inferred neuronal responses to humans averaged 0.361 ± 0.076, surpassing those to man‐made objects (0.343 ± 0.065), and were significantly higher than the responses to animals (overall 0.320 ± 0.073, with average signals for flying, terrestrial, aquatic, and insect categories being 0.302 ± 0.068, 0.332 ± 0.075, 0.298 ± 0.062, and 0.313 ± 0.068 respectively; all *p*‐values < 0.001, Figure 4E). These findings might offer additional computational support for the role of recognizing familiar stimuli in V1,^[^ ^18^ ^]^ as laboratory mice might be more attuned to human presence. The conclusions drawn from these predictions aligned with the analysis of the ground truth data, providing further validation for our findings derived from the predictions (Figure S2, Supporting Information).

### 2.4 The Computational Model Reveals Biological Spatial Properties of the Visual Cortex

By leveraging these predictions, we can computationally discover the general neuron organization in V1 and answer an important question of whether V1s of different mice respond to images similarly. Therefore, we analyzed the correlation between neuronal distances and prediction similarities within and across different brains. The similarity between neurons was quantified using correlation values derived from their predictions on 100 test images.

**Figure 5:**
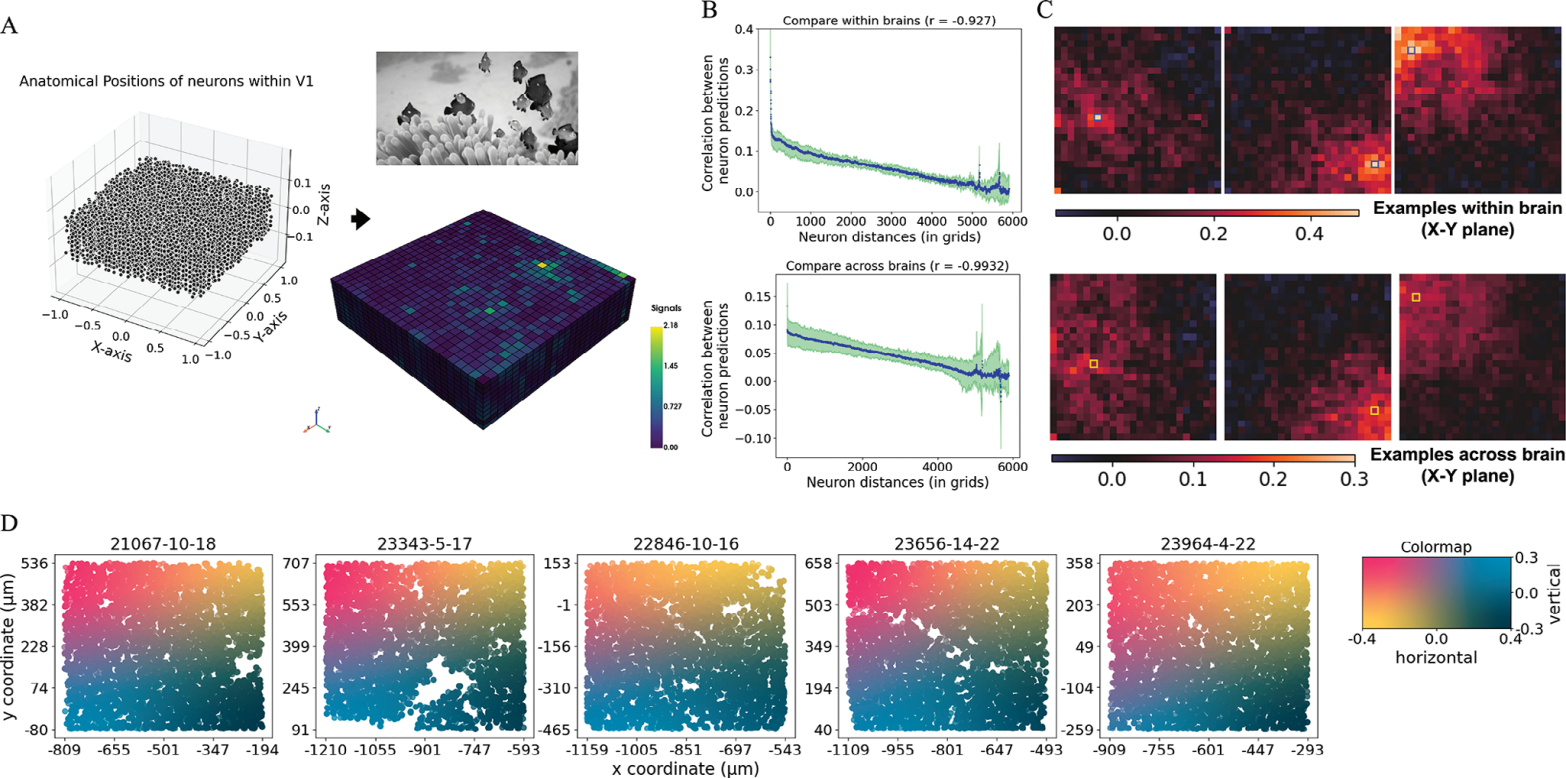
Analysis of the spatial properties from the neuron signal predictions with the grid‐based approach. A) describes the results after we grid the neurons. Each grid has 100 values interpolated from the predictions on the 100 test images. Here we show an example of the interpolations from the neuron predictions for a specific image. B) compares the neurons within and across brains, summarizing the general relationships between the neuron distances and the similarities among neuron activities. The blue dots are the averages across the brains or brain pairs, The light green areas cover the region between 5% and 95% percentiles of the original values. C) presents three examples for (B) with random reference grids labeled with blue (yellow) boxes for the comparisons within and across brains. The heatmaps summarize the distributions of the correlations to the reference grids on the X–Y plane. The visualized correlation scores were averaged along the Z‐axis (compare within the brain) and among the brain pairs (compare across brains). D) presents the retinotopic maps comparing the learned neuron receptive field (RF) centers with respect to the neuron anatomical coordinates (the x‐ and y‐axes). Each point represents a neuron, and the point color indicates its average RF center location on the latent visual representations (i.e., the core outputs) inferred from the readouts of ten models. The colormap (the right‐most panel) describes the mappings between the colors and relative locations on the latent representations. The relative locations range from −1 to 1 in both horizontal and vertical directions.

Given the variability in distances between neurons and the misalignment of positions across different brains, we employed a grid‐based approach to investigate the neurons' spatial characteristics.^[^ ^35^, ^36^ ^]^ We partitioned the regions with measured neurons in area V1 into 6250 evenly distributed grids with the shape of (25, 25, 10) along the X, Y, and Z axes. As the position range on Z was much smaller than the X's and Y's, the Z‐axis's partition number was the neuron locations' original layer number (**Figure** **5**A). The distance between grids was ≈0.08 on the X and Y axes and 0.015 on the Z‐axis.

We found a strong inverse relationship between the distance of neurons and signal similarity. For comparisons within the same brains, when inspecting brains separately or averaging across them, both results showed significant negative correlations between the similarities and distances, with correlation scores lower than −0.9 (Figure 5C, Figure S4A, Supporting Information). When comparing across different brains, the correlation between the neuron similarity and neuron distance remained significant (despite minor changes in the value of similarity between the closest neurons and the farthest neurons), suggesting the spatial organizations of V1 and visual reactions are conserved across mice (Figure 5B, Figure S5A, Supporting Information). This can be illustrated clearly when focusing on a specific, random reference neuron. Closer neurons around the reference behave more similarly than those located farther away (Figure 5C). Analyzing the recorded neuronal responses would retrieve similar results and conclusions regarding V1 spatial properties ( Figure S3A,B, Supporting Information).

The spatial arrangements of the neurons' receptive field (RF) centers within the latent visual representations (i.e., the core output) potentially explain the signal similarities. Correlating the RF center locations from the readout and the neuron anatomical coordinates aligned with the concept that neurons close in space will have similar receptive fields,^[^ ^37^ ^]^ which in turn, manifested as similar neuronal signaling patterns. Moreover, the spatial congruence remained consistent across brain regions, where neurons with analogous relative positions in separate brains exhibit comparable RF centers (Figure 5D, Figure S7, Supporting Information).

## 3 Discussion

In this study, we presented the top‐performing algorithm for predicting individual visual neuronal responses to natural stimuli. The model achieved significant improvements over the previous state‐of‐the‐art model in both the live test and final test sets, with improvements of ≈15% in terms of Correlation to Average ($\bar{R}$) and also showing considerable increases in the Fraction of Explainable Variance Explained (FEVE) scores by ≈30%.

Incorporating object position information is one of the key modifications to the input data. Its significant improvements indicate it potentially provides extra knowledge for predicting the neuron signals in pupil‐behavior‐free models. Assembling multiple models by averaging their predictions is another key step in accurately predicting neuronal signals. The final ensemble predictions benefit from a broader data spectrum compared to a single‐model approach by repetitively training the models on varying train‐validation splits. It allows the ensemble to effectively encapsulate a more comprehensive set of data features,^[^ ^38^ ^]^ significantly improving its performance. The enhanced accuracy observed in our ensemble model may echo the principles of population coding observed in the visual cortices, where integrated neuronal activities may achieve higher accuracy than single neurons in figure‐ground discrimination tasks.^[^ ^39^ ^]^

However, we recognized that neurons with low explainable variance (fraction explainable variance, FEVs) could be challenging for our models to predict, exhibiting limited improvements in accuracy. Neuron FEV was highly correlated with its performance (Pearson *R* = 0.624 for our method and 0.589 for the baseline model). Models only achieved modest accuracies on the neurons with FEVs less than 15%. Our method showed greater improvement on high‐FEV neurons than it did on low‐FEV ones (Figure S6, Supporting Information). Addressing the prediction of low‐FEV neurons with greater accuracy remains an open question for future investigation.

The predictions from our solution showed patterns consistent with in vivo studies, demonstrating the model's capability to assist in silico research in neuroscience. Exploring the influence of image properties on model predictions aligned our observations with the findings from previous studies, especially the asymmetric neuronal responses to dark and light stimuli. OFF responses were stronger than ON responses in V1 of various species, including the mouse.^[^ ^33^, ^34^, ^40^, ^41^ ^]^ Furthermore, the predicted neuron signal strengths varied depending on whether the images contained humans, animals, or man‐made objects, providing evidence that supports the notion that V1 plays a role in recognizing familiar stimuli.^[^ ^18^ ^]^ Our evaluation of other critical features, such as orientation selectivity,^[^ ^42^ ^]^ may be constrained by the complexity of the data. The orientation and the angle information in a natural image could be challenging to extract and study.

Leveraging this model, we described V1 spatial properties and answered whether V1 neurons from different mice would react similarly to the stimuli by finding that images elicit similar responses across different brains spatially in V1. By inspecting the locations of the neurons’ receptive field (RF) centers with respect to their anatomical coordinates, we showed that neurons close in space had similar RFs, which potentially accounted for the negative correlation between neuron distance and signal similarity, suggesting that neurons exhibiting similar behaviors are not randomly distributed but form local clusters within a brain. Furthermore, when analyzing the aligned neurons across brains, we observed similar patterns of RF center distributions and neuron distance‐signal relationships, suggesting that V1 had conserved (i.e., genetically encoded rather than learned) spatial organization for generating similar reactions to stimuli across mice.

Our model offered additional biological insights by estimating the artificial receptive fields (aRFs) through model predictions for each neuron^[^ ^43^ ^]^ (Supporting Information). Presenting the model with millions of white noise images and performing a weighted sum with respective neuron predictions, the estimated aRFs present clear on‐subregions and off‐subregions, which corresponds with properties of receptive fields found in vivo studies, as well as the region patterns for classifying simple neurons^[^ ^28^ ^]^ (Figure S8A, Supporting Information). 2D Gaussian fits on the aRFs suggested that their overall structures are similar across different mice and maintained the shape conformity with the receptive fields elucidated from the model's readout (Figure S8B,C, Supporting Information). Nonetheless, due to the absence of ground‐truth information on neuron types, further validation of the aRFs' estimations and exploration of the impact of neuron types on model predictions necessitate additional data (Figures S9, S10, Supporting Information).

## 4 Experimental Section

### Data Retrieval, Preprocessing, and Partition

The raw data can be accessed from https://gin.g‐node.org/cajal/Sensorium2022. Apart from the greyscale images and their corresponding neuron responses, the public dataset also provided an initial partition of the train, validation, live test, and final test, as well as the statistics of the images and the responses for each of the datasets, including the global mean ($\bar{I}$, $\bar{r}$) and standard deviation (*s_I_*, *s_r_*) from the training and validation data. $\bar{I}$ and *s_I_* were single values, while $\bar{r}$ and *s_r_* were vectors with lengths equal to the number of neuron responses. The raw images (*I*) were normalized by $\left(I-\bar{I}\right)/s_{I}$, which was the first channel of our inputs (Figure 2B), and the responses (*r*), both in the training and evaluation phase, were standardized by *r*/*s_r_*. The coordinates of the neurons were also normalized to (−1, 1) by first subtracting the average value of each axis and then dividing by the absolute maximum.

### Retrieve the Object Locations

The model for detecting objects in the grayscale images was created by fine‐tuning the pre‐trained YOLOv5‐large model on the ILSVRC dataset. We sampled 250 images for each category to form a 250 000‐image training set and used its 50 000‐image validation set for callback. All the images were converted to grayscale. We fine‐tuned the model with the parameters for low‐augmentation COCO training from scratch (https://github.com/ultralytics/yolov5/blob/master/data/hyps/hyp.scratch‐low.yaml) and modified the learning rate to 0.0005.

To make inferences on the SENSORIUM data, we used the pre‐trained YOLOv5‐large model to detect “human/person” and our fine‐tuned model to detect the other objects. The object confidence threshold and intersection over union (IoU) threshold were 0.5 and 0.5 for detecting “human/person”, and 0.05 and 0.5 for detecting the others. The image size given to the model was set to 256. The locations of the *i^th^* bounding box was represented by the XY‐axis of the bottom‐left corner and top‐right corner $\left[x_{i}^{L},\;y_{i}^{L},\;x_{i}^{R},\;y_{i}^{R}\right]$. For an image with *n* bounding boxes, we created a merged and padded box covering all objects:

(1) $$\begin{array}{ccc} & & [x^{L}=\;min\left(x_{1}^{L},\;\text{…},\;x_{n}^{L}\right),\;y^{L}=\;min\left(y_{1}^{L},\;\text{…},\;y_{n}^{L}\right),\;x^{R}\\ & & \;\;=\;max\left(x_{1}^{R},\;\text{…},\;x_{n}^{R}\right),\;\;y^{R}=\;max\left(y_{1}^{R},\;\text{…},\;y_{n}^{R}\right)] \end{array}$$

We then converted the box to a YOLO representation:

(2) $$\begin{array}{ccc} & & \left[x\;=\;\left(\left(x^{L}+x^{R}\right)/2\right)/256,\;y\;=\;\left(\left(y^{L}+y^{R}\right)/2\right)/144,\;w\;=\;\left(x^{R}-x^{L}\right)\right.\\ & & \;\;\left./\;256,\;h\;=\;\left(y^{R}-y^{L}\right)/144\right] \end{array}$$

256 and 144 were the respective image width and height. This representation provides a relative description of the image's bounding box position and size.

### Model Inputs

For a grayscale image *I* with a shape of 144 in height and 256 in width, together with its dataset global mean $\bar{I}$ and standard deviation *s_I_*, as well as the object bounding box [*x*,  *y*,  *w*,  *h*], we form the 6‐channel input including: (1) the normalized image $I_{norm}=\;\left(I-\bar{I}\right)/s_{I}$; (2) the centered image $I_{center}=\;\left(I_{norm}-{\bar{I}}_{norm}\right)$; (3‐6) the matrixes of the bounding box location x*1_144 × 256_, y*1_144 × 256_, w*1_144 × 256_, and h*1_144 × 256_, where 1_144 × 256_ represents the matrix of ones (Figure 2B).

### Model Structure

Our model structure was designed by implementing several improvements on Lurz et al.’s original work.^[^ ^15^ ^]^ It consisted of a convolutional neural network core for extracting the image information and a Gaussian readout for decoding the extracted features to the neuron responses (Figure 2B).

The core had five convolutional layers. The first layer was used for scaling the inputs, whose kernel size was four, the same as the strides. The last three layers were depth‐separable convolutions, and their outputs would have the same shapes as the inputs. Each of the five layers would be followed by a batch normalization layer and an ELU activation layer. The channel numbers were 32, 128, 256, 256, and 256.

The Gaussian readout generated inferences for each neuron through a linear combination of the feature vector from the core at a single spatial position, followed by an ELU activation with an offset of one to keep the inferences positive. The position was determined by a 2‐D Gaussian distribution whose mean μ_*i*_ represented the center of the receptive field of the neuron *i*, and standard deviation Σ_*i*_ represented the uncertainty of this field position. μ_*i*_ was learned from the coordinates of the neuron *i* through a readout position network, a fully connected network with one hidden layer and 30 hidden nodes. The input and hidden layers were activated by ELUs, and the output was activated by Tanh.

During training, the positions were randomly sampled from the receptive field. They became closer to the means as the standard deviations decreased over training. In the validation and testing phases, the positions were the means.

### Model Training

The model trained a shared core and separated readouts for seven mice to minimize the Poisson loss with the Adam optimizer with a batch size of 128:

(3) $$\frac{1}{m}\sum_{i=1}^{m}\left(o_{i}-r_{i}\log o_{i}\right)$$

*m* denoted the neuron number. *o_i_* and *r_i_* were single values representing the prediction and the ground truth of the neuron‐accumulated spiking activities responding to one stimulus in a batch. The learning rate was controlled by a scheduler implemented using the PyTorch function ReduceLROnPlateau, with the initial learning rate set at 0.009 and the minimum set at 0.0001. The scheduler reduced the learning rate by a factor of 0.3 when the improvements on the validation loss were less than 1e‐6 for more than five consecutive epochs. The validation loss was the correlation between the predictions and the ground truths. After at most four times rate decay or 200 epochs training, we saved the model having the best validation loss. Following the ensemble strategy, we repeated the entire training process ten times and saved ten separate models and their corresponding weights.

The weights and biases in the layers were initialized by the Xavier‐normal initialization and 0 s. The initial standard deviations were uniformly sampled from −0.1 to 0.1. The initial weights in the fully connected layer were 1/256 for linearly combining the feature vector from the core.

Each model was trained on a single Nvidia TITAN RTX GPU, and the average training time of an epoch was ≈1.5 min. All models would converge within 200 epochs.

### Model Ensemble

We created ten train‐validation splits for each mouse dataset based on the initial splits of training, validation, live test, and final test. Our splits randomly selected the same number of samples as the original validation set to form the new ones. Ten separate models on these splits are trained and averaged their predictions on the test data to form our final outputs (Figure 3D results). The results presented in Figure 3C were obtained by averaging the predictions among the first *N* models (*N* was the number on the x‐axis).

### Model Evaluation

The evaluation metrics in this challenge included the correlation to average and FEVE, the fraction explainable variance explained. correlations measured the neuron's relative response change but did not account for the unexplainable noise in neural response. FEVE, on the other hand, corrected for the unexplainable noise and provided an estimate for the upper bound of stimulus‐driven activity.

For each neuron, given its response *r_ij_* to the image *i* and repeat *j*, and the prediction *o_i_*, the Correlation to Average was computed between the prediction and the average neuron response $\bar{r_{i}}=\;\left(\sum_{j\;=\;1}^{J}r_{ij}\right)/J$ of the image *i* across the *J* repeats:

(4) $$\bar{R}=\frac{\sum_{i}\left({\bar{r}}_{i}-\bar{r}\right)\left(o_{i}-\bar{o}\right)}{\sqrt{\sum_{i}{\left({\bar{r}}_{i}-\bar{r}\right)}^{2}{\left(o_{i}-\bar{o}\right)}^{2}}}$$

where $\bar{r}$ and $\bar{o}$ were the average response and prediction across all repeats and images. Given the same information, the FEVE was computed as the following:

(5) $$1-\frac{\frac{1}{N}\sum_{i,j}{\left(r_{ij}-o_{i}\right)}^{2}-\sigma_{\varepsilon }^{2}}{\mathrm{Var}\left[r\right]-\sigma_{\varepsilon }^{2}}$$

where Var[**r**] referred to the total variance computed across all N trials, and $\sigma_{\varepsilon }^{2}$ denoted the unexplainable noise computed from $(\sum_{i\;=\;1}^{I}Var[r_{i}])/I$. Var[r_i_] was the variance of the responses to the image *i*. As the neurons with low explainable variance can have a substantially negative FEVE value and bias the population average, the calculations were restricted to neurons with fraction explainable variances (FEVs) larger than 15%.^[^ ^7^ ^]^ FEVs were generated by:

(6) $$\frac{\mathrm{Var}\left[r\right]-\sigma_{\varepsilon }^{2}}{\mathrm{Var}[r]}$$

### Image Properties Extraction

We extracted three types of properties from the images (Figure 4A), including the complexity quantified in edge magnitudes, image brightness, and object classes. We manually labeled the test images into four general categories and further split the animals into another four types. Figure S1 (Supporting information) shows some examples of these categories. The brightnesses were the average pixel values of the grayscale image, and the contrasts were the standard deviations. The method for quantifying the complexity referred to the spatial information^[^ ^31^ ^]^ and used the functions from OpenCV. Let *s_v_* and *s_h_* represent the grayscale images filtered by the vertical and horizontal Sobel kernels. The spatial information was $SI\;=\sqrt{s_{v}^{2}+s_{h}^{2,}}\;$ and the complexity was the average *SI* across all pixels.

### Grids Generation, Interpolation, and Analysis

*Generate grids and interpolate*: The grid coordinates on the X and Y axes were 25 points evenly distributed between −1 and 1, while the coordinates on the Z‐axis were ten points evenly distributed between the maximum and minimum values among the dataset from the 5 mouse brains. We interpolated the grid values by utilizing the “griddata” function from SciPy with the linear method.

*Patterns between neuron similarity and distance within and across the brain*: To summarize the changes of the neuron similarities against the neuron distance within the brain, we first removed the grids that contain NaNs, sorted them by their distances to the first grid (the bottom‐left one), and then calculated the correlations among them, which form a matrix. In the correlation matrix, the k‐th off‐diagonal indicated the correlations between the two neurons with a k distance. Figure 5C plots the k against the average correlation values on the k‐th off‐diagonal. When summarizing the changes across brains, the difference was that we only kept the grids that did not contain NaNs in both brains.

*Inspect specific reference grids*: To visualize the correlation distribution among random reference grids, we calculated the correlations to the reference grids in different directions, plotting the heatmaps as the orthographic projection. For example, when we inspected the correlation distribution on the X‐Y plane comparing within brains, for each brain, we first generated the correlations for each of the Z and then averaged them with the “nanmean” function from the NumPy library, which can handle the NaNs. Inspections on the X‐Z and Y‐Z planes were the same. Figure 5C, left panel, shows the “nanmean” average distribution from the five mice. As for the comparison across brains, in Figure 5C, right panel, we repeated the above pipeline for each brain pair and then averaged the distributions from ten pairs.

### Statistical Test

All of the claims of significance were based on statistical hypothesis testing. The P‐values reported with the correlations between neuron signal predictions and image properties (Figure 4C,D) were retrieved from the correlation test using the “cor.test” function in R and the “pearsonr” function from SciPy. The P‐values for comparing the neuron signals to respond to different types (Figure 4E) of objects were retrieved from the Wilcoxon tests using the “wilcox.test” function in R.

### Code Availability

The code for our model and analysis can be found at https://github.com/GuanLab/Sensorium2022_Challenge/. The code for fine‐tuning the object detection model is from PyTorch: https://github.com/ultralytics/yolov5.

## Conflict of Interest

The authors declare no conflict of interest.

## Supporting information

Supporting Information

## Data Availability

The raw data of the SENSORIUM can be retrieved from https://gin.g‐node.org/cajal/Sensorium2022. The data of ILSVRC can be found at https://www.kaggle.com/c/imagenet‐object‐localization‐challenge.
