# Supplementary material for: Predicting Single Neuron Responses of the Primary Visual Cortex with Deep Learning Model
Source: Adv Sci (Weinh). 2024 Feb 13;11(15):2305626. doi: 10.1002/advs.202305626 (PMC11022733; doi:10.1002/advs.202305626)

ADVANCED SCIENCE
Open Access

Supporting Information

for *Adv. Sci.*, DOI 10.1002/advs.202305626

Predicting Single Neuron Responses of the Primary Visual Cortex with Deep Learning Model

*Kaiwen Deng, Peter S. Schwendeman and Yuanfang Guan**

Supplementary Methods

Generate artificial receptive fields

We first drew N = 2,000,000 white noise images from a uniform distribution and presented them to the trained model. The aRF of the neuron *i* will be the summation of all noise images weighted by the respective prediction.

$$\mathrm{aRF_i} = \sum_{\mathrm{n}}^{\mathrm{N}} \mathrm{F(x_n)} * \mathrm{x_n} \quad \mathrm{x_n} \sim \mathcal{U}^{1\times144\times256}$$

The x_n denotes the 1-channel noise image with a size of 144 by 256. The **F** represents the whole inference procedure of our model, including the data preprocessing steps for forming the 6-channel input and the ensemble. We set the last 4 channels (x, y, w, h) as (0.5, 0.5, 1, 1) for the noise image inputs.

To quantify the locations and sizes of the aRFs, a 2D Gaussian was fit to each aRF. Concretely, each aRF was first normalized by subtracting the mean and dividing the standard deviation from all aRFs, then taking their absolute values and fitting a 2D Gaussian using the "curve_fit" function from SciPy. As not all aRFs had good fits, the bottom 5% with too large sigmas were dropped.

Supplementary Figure 1: Examples of the manually categorized images. If the image contains multiple classes of objects, it will be categorized into these classes multiple times.

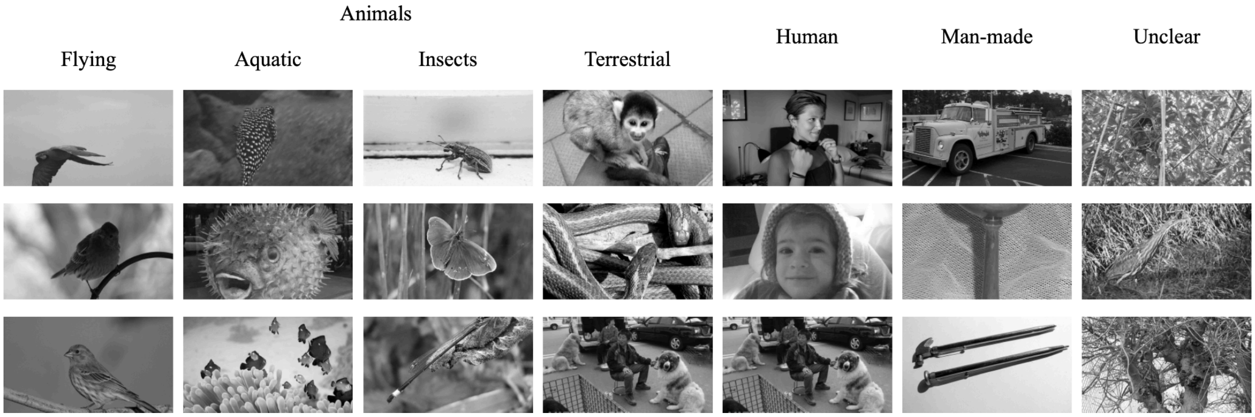

Supplementary Figure 2: The image property and content effects on the predicted and ground-truth neuron signals corresponding to Figure 4. (A) summarize the correlations among the image properties: complexity, brightness, and contrast, and the predicted neuron signals, when (B) summarizes these relationships on the ground-truths. (C) illustrates the relationship in (B) with a graph, and (D) shows the content effects.

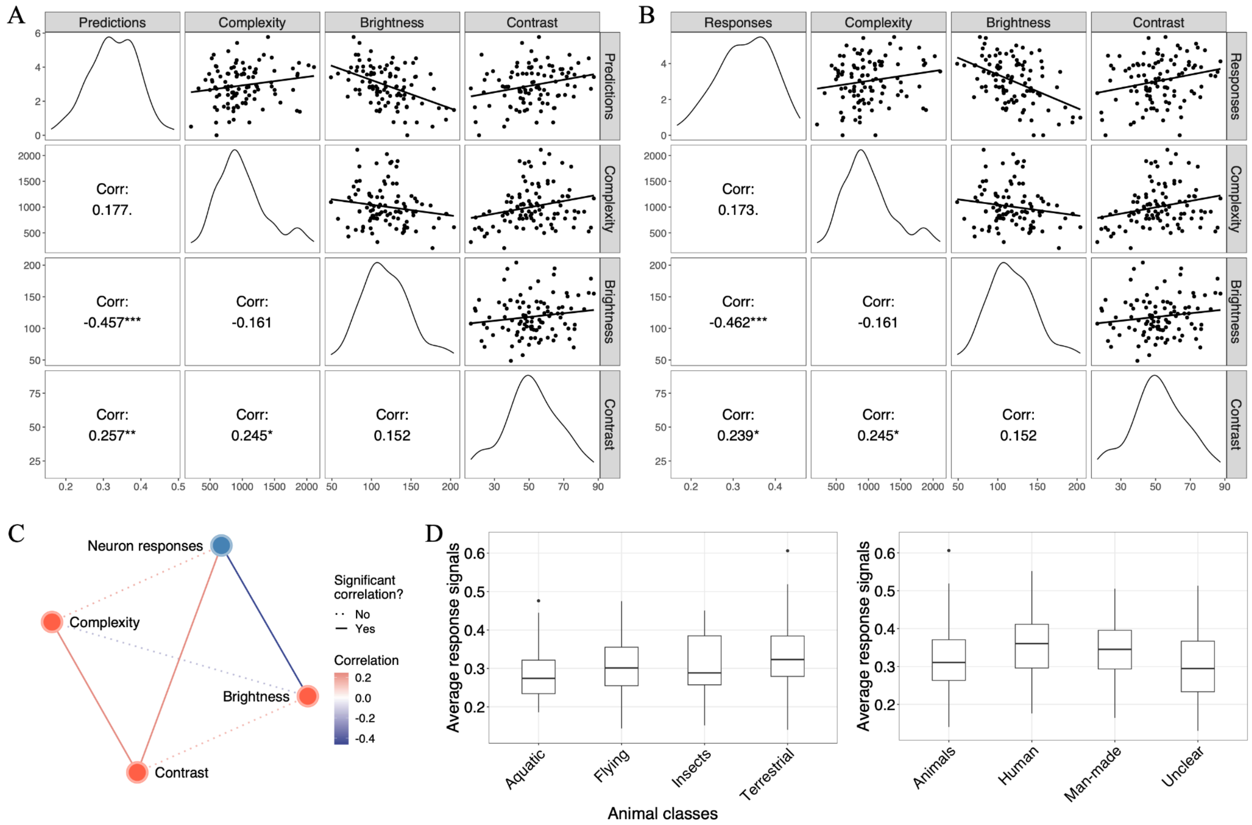

Supplementary Figure 3: Analyses of the V1 spatial properties on the neuron responses, compared to Figure 5, which used the neuron predictions. (A) describes the overall signal correlation changes along with the neuron distance in the within-brain and cross-brain comparisons. (B) show the heatmaps illustrating the signal correlations to a specific reference grid viewing on the X-Y plane from the comparisons within and across brains.

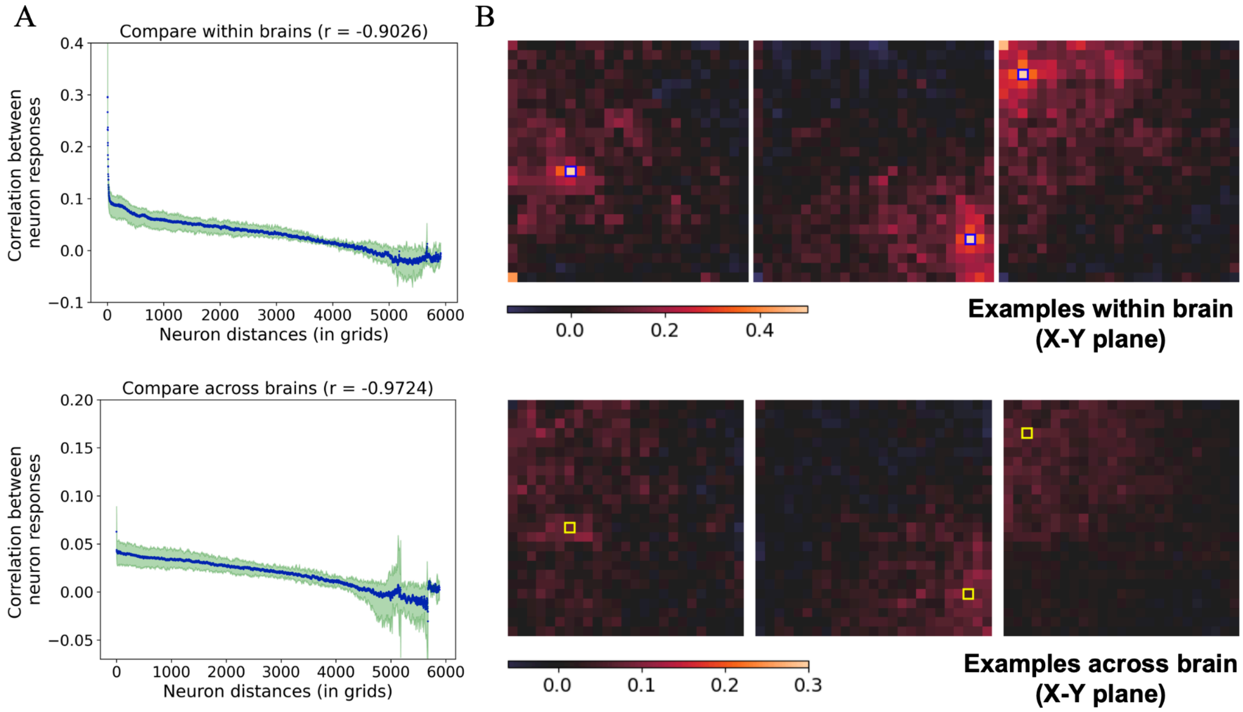

Supplementary Figure 4: The relationships between the neuron signal similarity and the neuron distance within the brains. (A) shows the relationships of the neuron signal predictions for each brain, while (B) shows the ground-truth neuron responses.

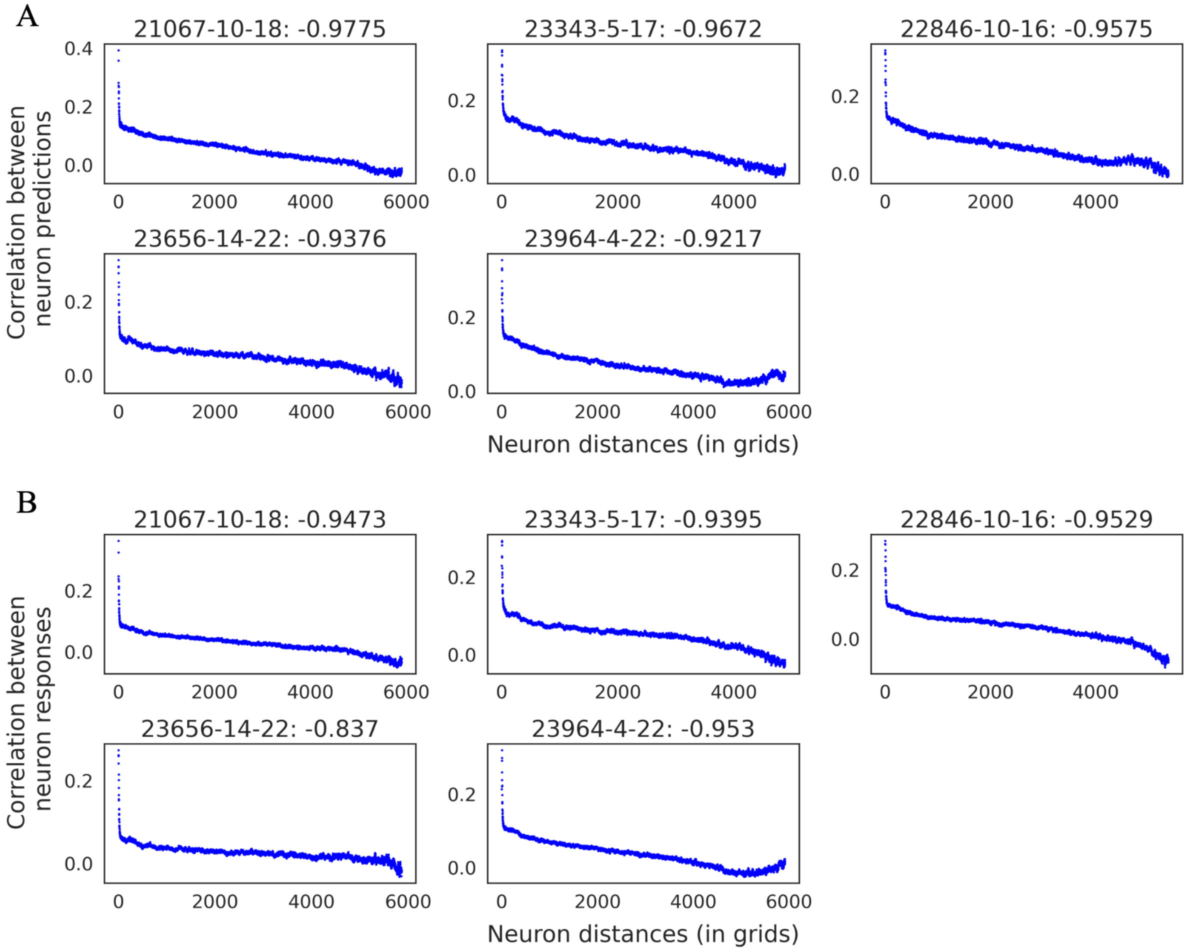

Supplementary Figure 5: The relationships between the neuron signal similarity and the neuron distance across the brains. (A) shows the relationships of the neuron signal predictions for each pair of brains, while (B) shows the ground-truth neuron responses.

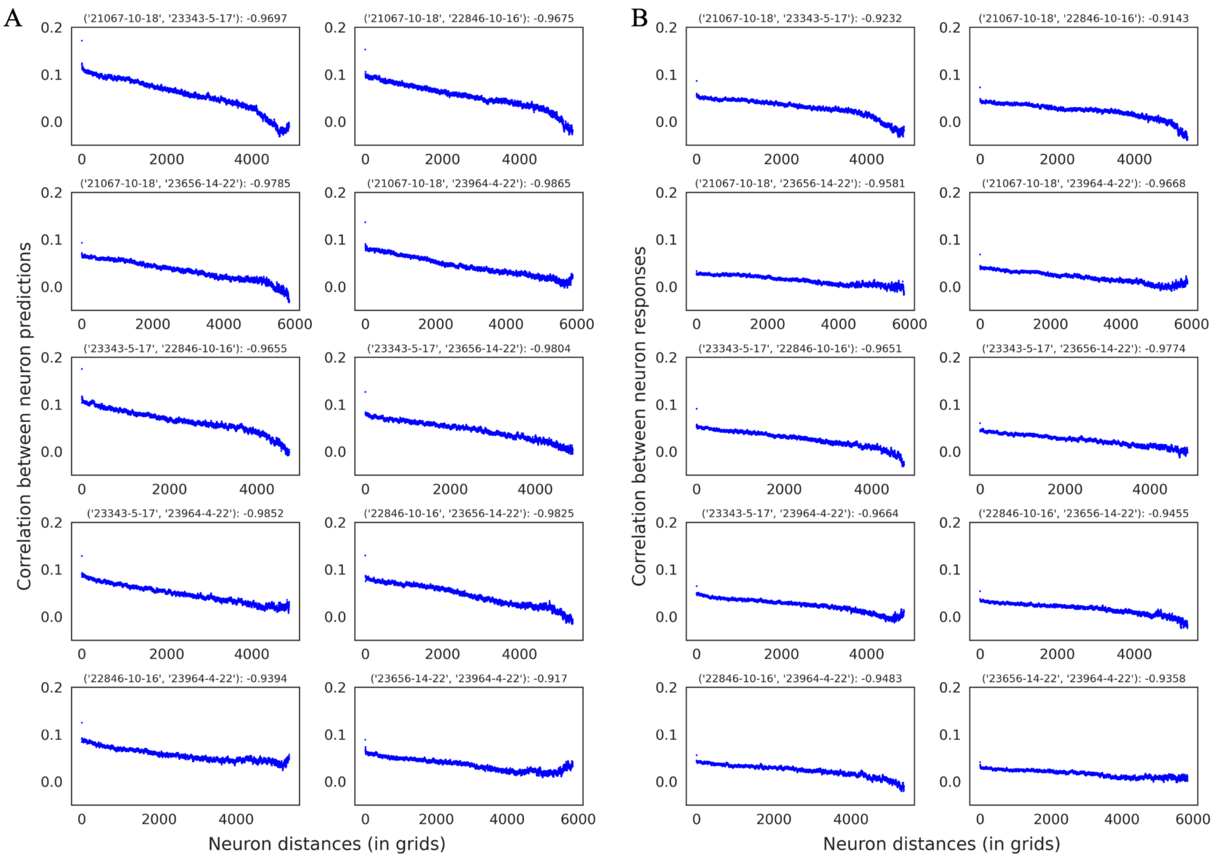

Supplementary Figure 6: The relationships between the fraction explainable variance (FEVs) and the model performances for each neuron. (A) shows the relationships of all neurons and compares the baseline model and our method. (B) shows the lines fit on the neurons in (A) using the geom_smooth function from the ggplot2 package with the default parameters.

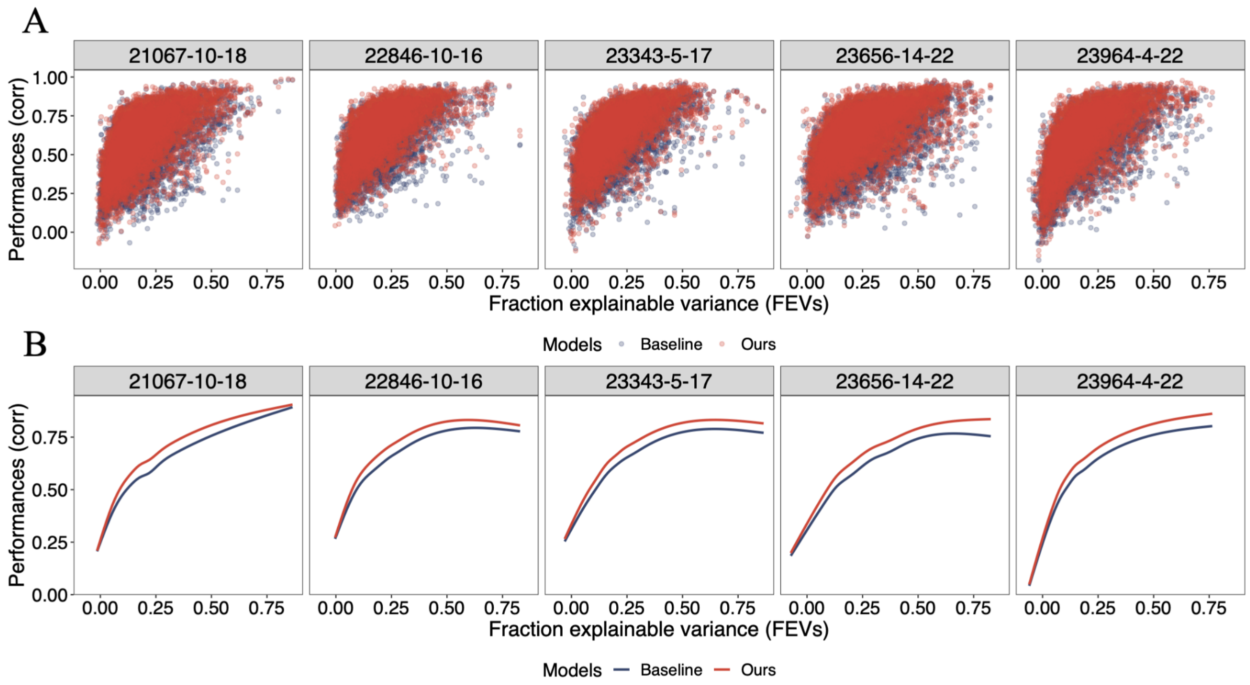

Supplementary Figure 7: The neurons' learned receptive field centers on the core's latent representations. The x- and y-axes are the relative positions of the latent features, ranging from -1 to 1. Each point represents a neuron.

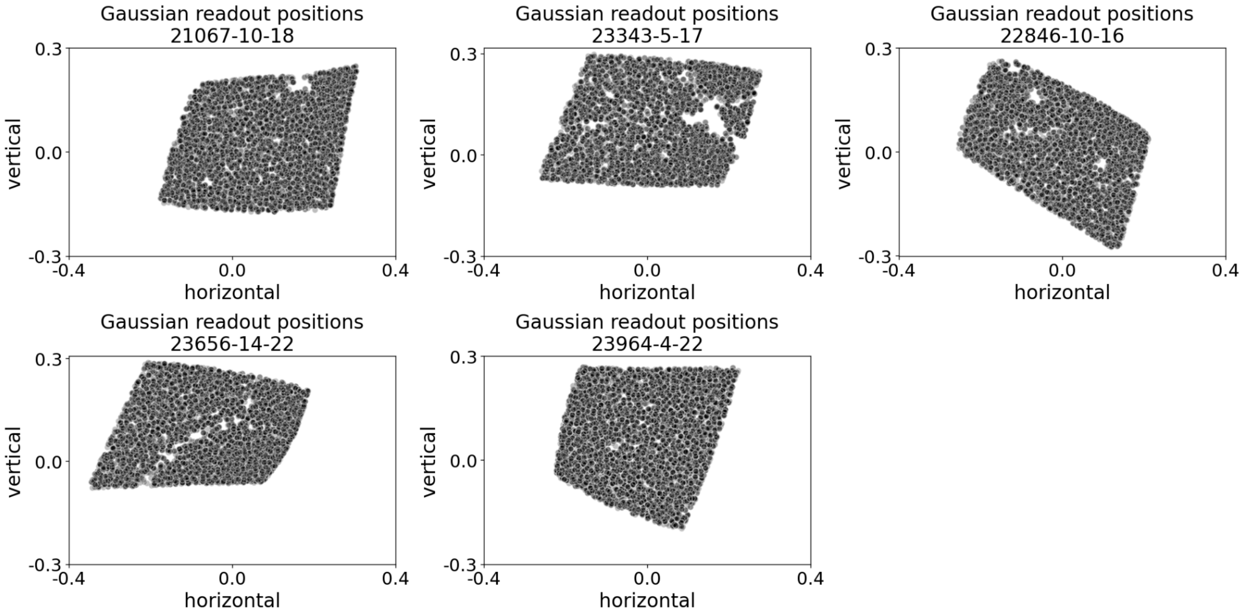

Supplementary Figure 8: The neurons' artificial receptive fields (aRFs) structures. (A) visualized the aRFs of 6 random neurons from 2 mice. Darker and lighter regions may reflect the off-subregions and the on-subregions. (B) is a KDE plot visualizing the distribution of the aRFs centers from the 2D Gaussian fits and (C) shows the distribution of the $\boldsymbol{\sigma}$.

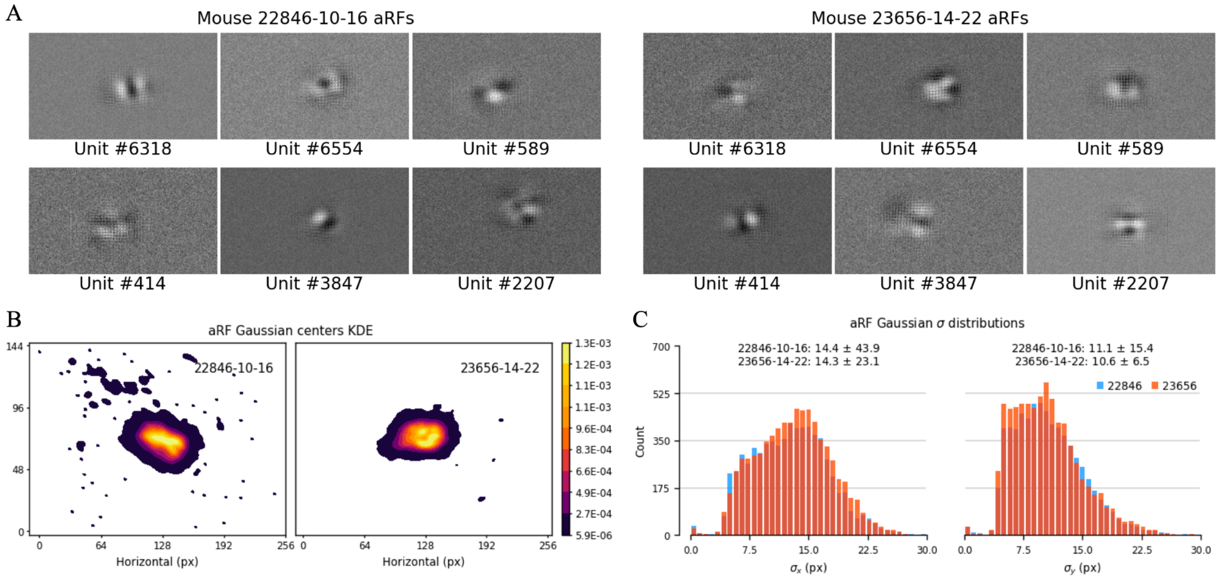

Supplementary Figure 9: 70 neurons sampled among the top-performing and bottom-performing neurons from mouse 23656-14-22.

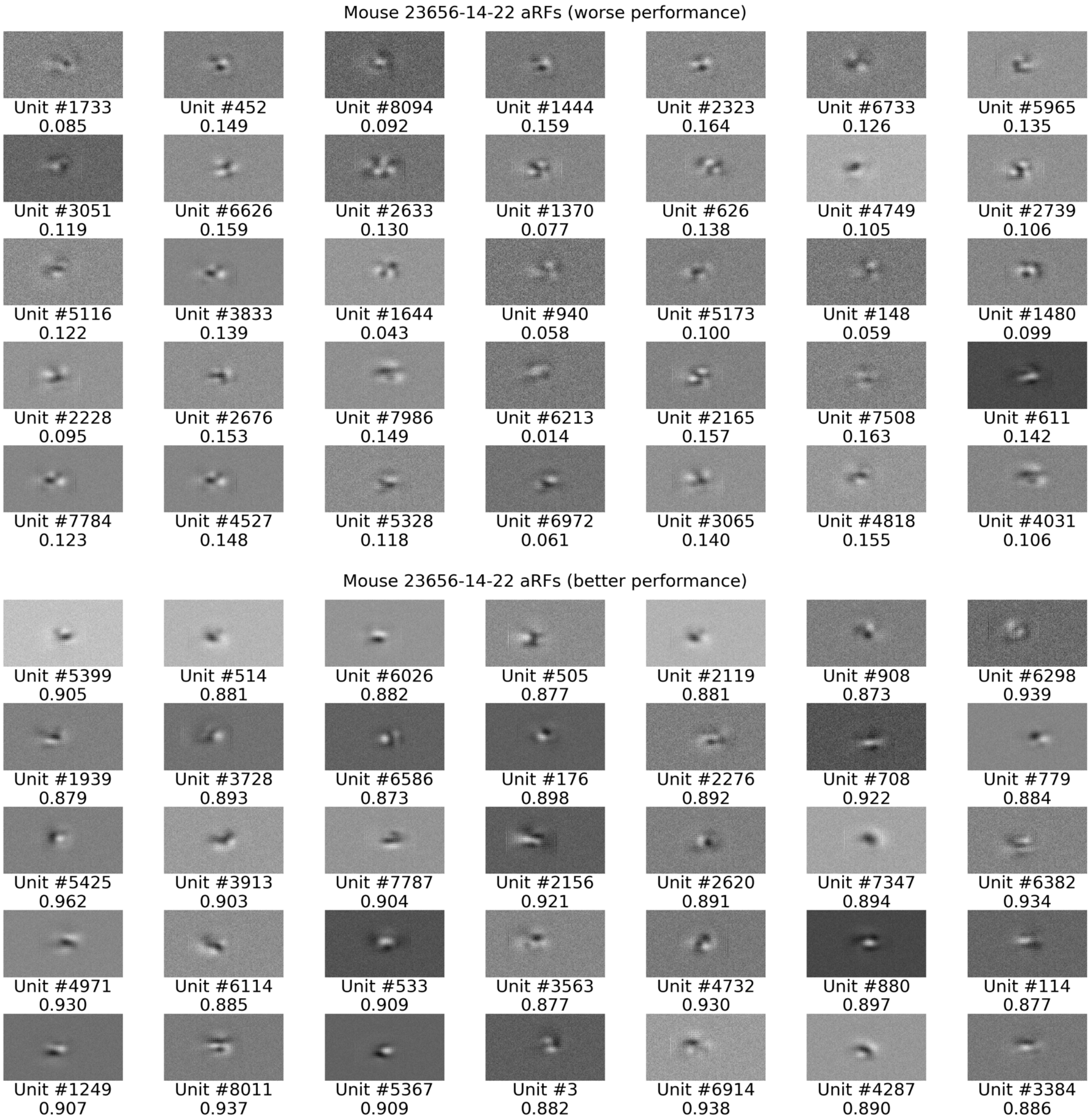

Supplementary Figure 10: 70 neurons sampled among the top-performing and bottom-performing neurons from mouse 22846-10-16.

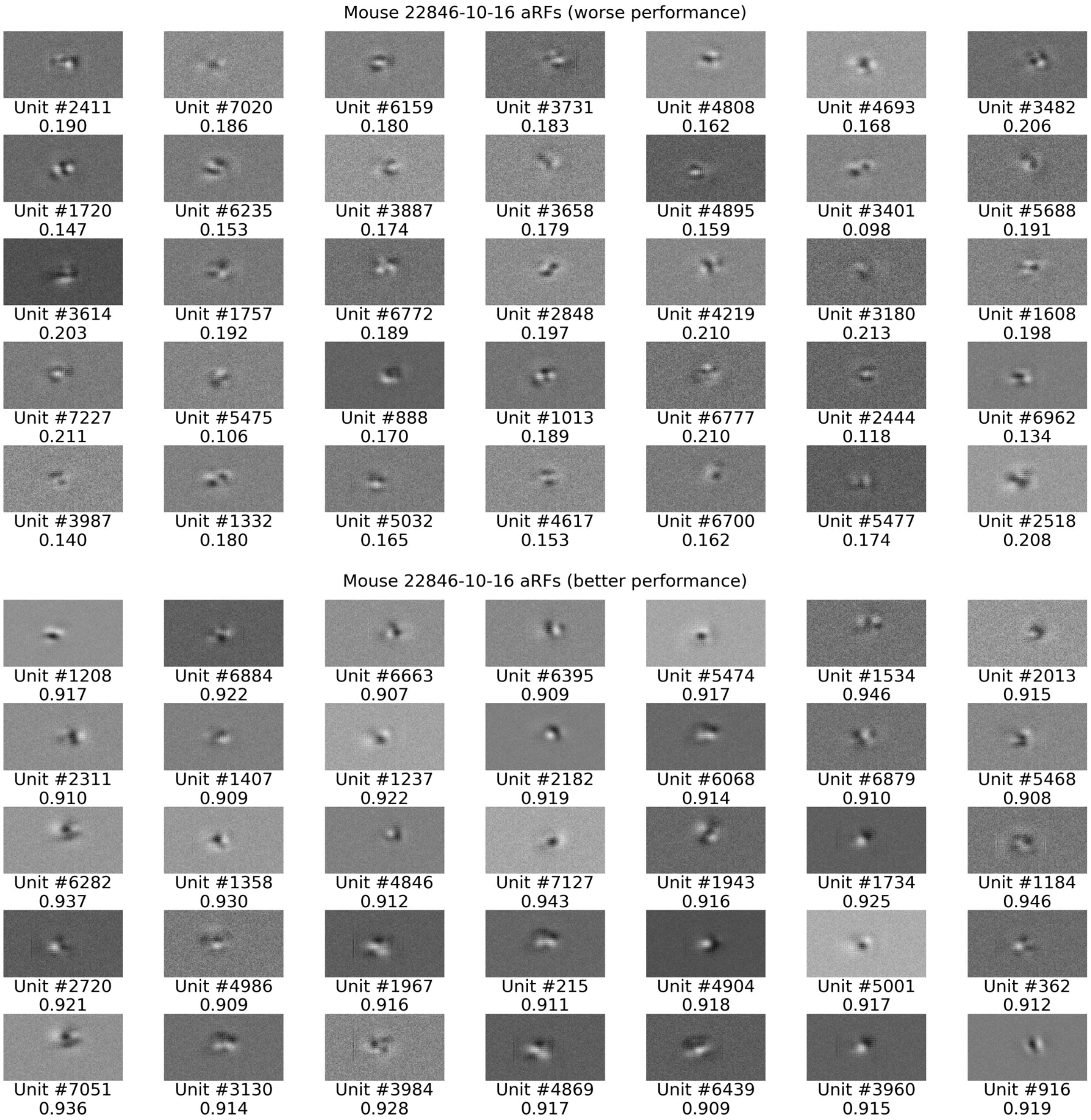

Supplement: Supplementary file 1 — Supporting Information [file ADVS-11-2305626-s001.pdf]
